# Sex differences in the clinical manifestation of autosomal dominant frontotemporal dementia

**DOI:** 10.1002/alz70857_100820

**Published:** 2025-12-24

**Authors:** Kaitlin B Casaletto

**Affiliations:** ^1^ Memory and Aging Center, UCSF Weill Institute for Neurosciences, University of California, San Francisco, San Francisco, CA, USA

## Abstract

**Background:**

Sex differences are apparent in neurodegenerative diseases, but have not been comprehensively characterized in frontotemporal dementia (FTD).

**Method:**

Participants included 337 adults with autosomal dominant FTD enrolled in the ALLFTD Consortium. Clinical assessments and plasma were collected annually for up to six years. Disease stage was operationalized by the FTLD‐CDR (0=asymptomatic, 0.5=MCI, ≥1=dementia). Linear mixed‐effects models investigated how sex and disease stage associated with longitudinal trajectories of cognition, function, and neurofilament light chain (NfL).

**Result:**

Of 337 participants (99 *MAPT*, 83 *GRN*, and 191 *C9orf72)*, 204 (61%) were female and mean age was 50.64 (14.4) years. 185 (50%) were symptomatic at baseline. Whereas genotype, baseline demographics, and most clinical outcomes did not statistically differ between sexes, females demonstrated higher baseline NfL than males, and more rapid NfL accumulation over time regardless of disease stage. While sex differences were not apparent at asymptomatic stages, females showed more rapid declines across all outcomes in symptomatic stages compared to males. Among asymptomatic participants, the association between baseline NfL and clinical trajectories was weaker in females versus males, a difference that attenuated in symptomatic participants.

**Conclusion:**

In genetic FTD, females show cognitive resilience in early disease stages followed by steeper clinical declines later in disease. Baseline NfL may be a less sensitive prognostic tool for clinical progression in females with FTD‐causing mutations, especially early in disease.

